# Trineodymium(III) penta­iron(III) dodeca­oxide, Nd_3_Fe_5_O_12_

**DOI:** 10.1107/S1600536809036794

**Published:** 2009-09-26

**Authors:** Takashi Komori, Terutoshi Sakakura, Yasuyuki Takenaka, Kiyoaki Tanaka, Takashi Okuda

**Affiliations:** a Graduate School of Materials Science and Engineering, Nagoya Institute of Technology, Gokiso-cho, Showa-ku, Japan; b Hokkaido University of Education HAKODATE, Yahata-cho, Hakodate-shi, Japan

## Abstract

The title compound, Nd_3_Fe_5_O_12_ (NdIG), has an iron garnet structure. One of the Fe atoms is coordinated by six O atoms in a slightly distorted octa­hedral geometry and has 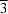 site symmetry. The other Fe atom is coordinated by four O atoms in a slightly distorted tetra­hedral geometry and has 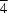 site symmetry. The FeO_6_ octa­hedron and FeO_4_ tetra­hedron are linked together by corners. The Nd atom is coordinated by eight O atoms in a distorted dodeca­hedral geometry and has 222 site symmetry. The O atoms occupy general positions.

## Related literature

The title compound is isotypic with the *Ia* 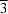 *d* form of Y_3_Fe_5_O_12_ (YIG), see: Bonnet *et al.* (1975 ▶). For crystal growth from low-temperature liquid-phase epitaxy, see: Fratello *et al.* (1986 ▶). X-ray intensities were measured avoiding multiple diffraction, see: Takenaka *et al.* (2008 ▶). For details of the full-matrix least-squares program *QNTAO*, see: Tanaka *et al.* (2008 ▶). For the anisotropic extinction refinement, see: Becker & Coppens (1975 ▶).

## Experimental

### 

#### Crystal data

Nd_3_Fe_5_O_12_
*M* *_r_* = 903.97
Cubic, 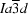

*a* = 12.6128 (2) Å
*V* = 2006.48 (6) Å^3^
*Z* = 8
Synchrotron radiation
λ = 0.67171 Å
μ = 18.30 mm^−1^
*T* = 298 K
0.025 mm (radius)

#### Data collection

Rigaku AFC four-circle diffractometer
Absorption correction: spherical [transmission coefficients for spheres tabulated in International Tables C (1992 ▶), Table 6.3.3.3, were interpolated with Lagrange’s method (four point interpolation; Yamauchi *et al.*, 1965 ▶)] *T*_min_ = 0.502, *T*_max_ = 0.527
6653 measured reflections
1159 independent reflections
1159 reflections with *F* > 3σ(*F*)
*R*_int_ = 0.017

#### Refinement

*R*[*F* ^2^ > 2σ(*F* ^2^)] = 0.016
*wR*(*F* ^2^) = 0.018
*S* = 1.42
6653 reflections
23 parameters
Δρ_max_ = 1.61 e Å^−3^
Δρ_min_ = −1.75 e Å^−3^

### 

Data collection: *AFC-5*, specially designed for PF-BL14A (Rigaku Corporation, 1984 ▶) and *IUANGLE* (Tanaka *et al.*, 1994 ▶).; cell refinement: *RSLC-3* (Sakurai & Kobayashi, 1979 ▶); data reduction: *RDEDIT* (Tanaka, 2008 ▶); program(s) used to solve structure: *QNTAO* (Tanaka *et al.*, 2008 ▶); program(s) used to refine structure: *QNTAO* (Tanaka *et al.*, 2008 ▶); molecular graphics: *ATOMS for Windows* (Dowty, 2000 ▶); software used to prepare material for publication: *RDEDIT*.

## Supplementary Material

Crystal structure: contains datablocks global, I. DOI: 10.1107/S1600536809036794/br2118sup1.cif

Structure factors: contains datablocks I. DOI: 10.1107/S1600536809036794/br2118Isup2.hkl

Additional supplementary materials:  crystallographic information; 3D view; checkCIF report

## Figures and Tables

**Table:** 

Nd1—O1	2.41820 (10)
Nd1—O1^i^	2.52960 (10)
Fe1—O1	2.03300 (10)
Fe2—O1^ii^	1.87550 (10)

**Table:** 

O1—Fe1—O1^i^	85.59 (1)
O1^ii^—Fe2—O1^iii^	114.47 (1)
O1^ii^—Fe2—O1^iv^	99.87 (1)

Symmetry codes: (i) 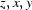; (ii) 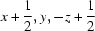; (iii) 
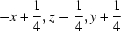
; (iv) 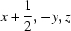.

**Fig. 1.**
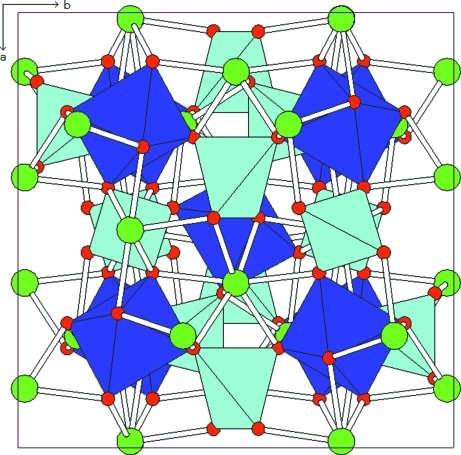
The structure of Nd3Fe5O12. Small red and large green spheres represent O and Nd atoms, respectively. Purple octahedron and blue tetrahedron represent FeO6 and FeO4 units, respectively.

**Fig. 2.**
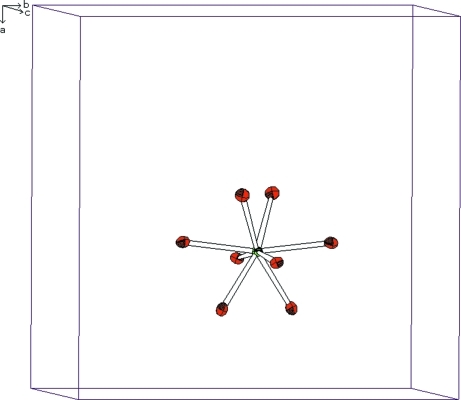
View of NdO8 with displacement ellipsoids at the 90% probability level. Green and red ellipsoids represent Nd and O atoms, in Fig.1.

## supplementary crystallographic information

### Comment

The title compound, Nd_3_Fe_5_O_12_ (NdIG), was difficult to be grown. It was grown by the low-temperature-liquid-phase epitaxy for the first time by Fratello *et al.* (1986). Though the crystal structure was assumed as iron-garnet-type structure by lattice constant and extinction rule, the complete structure was not determined. In this paper, we determine the O atom position and the complete structure by the full matrix least-squares program QNTAO. Since the R-factor is small and the residual density has no significant peaks where no atoms exists, the structure was finally determined to be iron-garnet structure. It is isotypic with the Ia3d form of Y_3_Fe_5_O_12_ (YIG). (Bonnet *et al.*, 1975). The Nd atom is coordinated by eight oxygen atoms. It forms a distorted dodecahedron. There are two Fe site symmetries. One of the Fe atom is coordinated by six oxygen atoms with site symmetry 3. It forms a slightly distorted octahedron. The other Fe atom is coordinated by four oxygen atoms, site symmetry 4. It forms a slightly distorted tetrahedron. FeO_6_ octahedron and FeO_4_ tetrahedron are linked together by corners. The structure of NdIG is drawn in Fig.1. And displacement ellipsoids of NdO_8_ is drawn in Fig.2.

### Experimental

Single crystals of neodymium iron garnet were prepared by low temperature liquid phase epitaxy on Sm_3_(ScGa)_5_O_12_ seeds with lattice parameters near the projected values for NdIG.

### Refinement

The Becker–Coppens type 1 Gaussian anisotropic extinction parameters were employed (× 10^-4^ seconds). z11 = 10.2 (5), z22 = 10 (2), z33 = 12 (2), z12 = 1(1), z13 = -0.5 (7), z23 = -1(1). X-ray intensities were measured avoiding multiple diffraction. (Takenaka *et al.*,  2008).

### Crystal data

**Table:**

Nd_3_Fe_5_O_12_	*D*_x_ = 5.985 Mg m^−^^3^
*M**_r_* = 903.97	Synchrotron radiation, λ = 0.67171 Å
Cubic, *I**a*3*d*	Cell parameters from 24 reflections
Hall symbol: -I 4bd 2c 3	θ = 35.7–42.4°
*a* = 12.6128 (2) Å	µ = 18.30 mm^−^^1^
*V* = 2006.48 (6) Å^3^	*T* = 298 K
*Z* = 8	Sphere, black
*F*(000) = 3248	0.03 mm (radius)

### Data collection

**Table:**

Rigaku AFC four-circle diffractometer	1159 independent reflections
Si 111	1159 reflections with *F* > 3σ(*F*)
Detector resolution: 1.25×1.25 degrees pixels mm^-1^	*R*_int_ = 0.017
ω/2θ scans	θ_max_ = 53.9°, θ_min_ = 3.7°
Absorption correction: for a sphere Transmission coefficients for spheres tabulated in International Tables C (1992\bbr00), Table 6.3.3.3, were interpolated with Lagrange's method (four point interpolation; Yamauchi *et al.*, 1965).	*h* = −8→30
*T*_min_ = 0.502, *T*_max_ = 0.527	*k* = −8→30
6653 measured reflections	*l* = −8→30

### Refinement

**Table:**

Refinement on *F*	Primary atom site location: isomorphous structure methods
Least-squares matrix: full	Weighting scheme based on measured s.u.'s
*R*[*F*^2^ > 2σ(*F*^2^)] = 0.016	(Δ/σ)_max_ = 0.003
*wR*(*F*^2^) = 0.018	Δρ_max_ = 1.61 e Å^−^^3^
*S* = 1.42	Δρ_min_ = −1.75 e Å^−^^3^
6653 reflections	Extinction correction: (B-C type 1 Gaussian anisotropic; Becker & Coppens (1975)
23 parameters	Extinction coefficient: 0.308 (5)

### Fractional atomic coordinates and isotropic or equivalent isotropic displacement parameters (Å^2^)

**Table:**

	*x*	*y*	*z*	*U*_iso_*/*U*_eq_	
Nd1	0.125000	0.000000	0.250000	0.00557 (1)	
Fe1	0.000000	0.000000	0.000000	0.00501 (1)	
Fe2	0.375000	0.000000	0.250000	0.00564 (1)	
O1	−0.029295 (2)	0.053092 (2)	0.149342 (2)	0.00762 (5)	

### Atomic displacement parameters (Å^2^)

**Table:**

	*U*^11^	*U*^22^	*U*^33^	*U*^12^	*U*^13^	*U*^23^
Nd1	0.00421 (1)	0.00525 (1)	0.00525 (1)	0	0	0.00121 (1)
Fe1	0.00501 (2)	0.00501 (2)	0.00501 (2)	−0.00024 (2)	−0.00024 (2)	−0.00024 (2)
Fe2	0.00442 (3)	0.00625 (2)	0.00625 (2)	0	0	0
O1	0.00791 (8)	0.00880 (9)	0.00614 (7)	−0.00027 (7)	0.00102 (6)	0.00041 (7)

### Geometric parameters (Å, °)

**Table:**

Nd1—O1	2.41820 (10)	Fe1—O1^i^	2.03300 (10)
Nd1—O1^i^	2.52960 (10)	Fe1—O1^viii^	2.03300 (10)
Nd1—O1^ii^	2.41820 (10)	Fe1—O1^ix^	2.03300 (10)
Nd1—O1^iii^	2.52960 (10)	Fe1—O1^x^	2.03300 (10)
Nd1—O1^iv^	2.41820 (10)	Fe1—O1^xi^	2.03300 (10)
Nd1—O1^v^	2.52960 (10)	Fe2—O1^xii^	1.87550 (10)
Nd1—O1^vi^	2.41820 (10)	Fe2—O1^iv^	1.87550 (10)
Nd1—O1^vii^	2.52960 (10)	Fe2—O1^xiii^	1.87550 (10)
Fe1—O1	2.03300 (10)	Fe2—O1^vi^	1.87550 (10)
			
O1—Nd1—O1^i^	67.83 (1)	O1—Fe1—O1^viii^	85.59 (1)
O1—Nd1—O1^ii^	72.82 (1)	O1—Fe1—O1^ix^	180.00
O1—Nd1—O1^iii^	124.94 (1)	O1—Fe1—O1^x^	94.41 (1)
O1—Nd1—O1^iv^	110.91 (1)	O1—Fe1—O1^xi^	94.41 (1)
O1—Nd1—O1^v^	72.97 (1)	O1^xii^—Fe2—O1^vi^	114.47 (1)
O1—Nd1—O1^vi^	159.79 (1)	O1^xii^—Fe2—O1^iv^	114.47 (1)
O1—Nd1—O1^vii^	95.60 (1)	O1^xii^—Fe2—O1^xiii^	99.87 (1)
O1—Fe1—O1^i^	85.59 (1)		

Symmetry codes:  (i) *z*, *x*, *y*; (ii) *x*, −*y*, −*z*+1/2; (iii) *z*, −*x*, −*y*+1/2; (iv) −*x*+1/4, −*z*+1/4, −*y*+1/4; (v) −*z*+1/4, −*y*+1/4, −*x*+1/4; (vi) −*x*+1/4, *z*−1/4, *y*+1/4; (vii) −*z*+1/4, *y*−1/4, *x*+1/4; (viii) *y*, *z*, *x*; (ix) −*x*, −*y*, −*z*; (x) −*z*, −*x*, −*y*; (xi) −*y*, −*z*, −*x*; (xii) *x*+1/2, *y*, −*z*+1/2; (xiii) *x*+1/2, −*y*, *z*.
